# The *Drosophila* TRPA1 Channel and Neuronal Circuits Controlling Rhythmic Behaviours and Sleep in Response to Environmental Temperature

**DOI:** 10.3390/ijms18102028

**Published:** 2017-10-03

**Authors:** Sanne Roessingh, Ralf Stanewsky

**Affiliations:** 1 Department of Cell and Developmental Biology, University College London, London WC1E 6DE, UK; s.roessingh@ucl.ac.uk; 2 School of Biological and Chemical Sciences, Queen Mary University of London, London E1 4NS, UK

**Keywords:** TRP channels, *Drosophila*, circadian clock, sleep, siesta, neuronal circuits, temperature sensing, temperature preference, temperature synchronisation, temperature entrainment

## Abstract

*trpA1* encodes a thermosensitive transient receptor potential channel (TRP channel) that functions in selection of preferred temperatures and noxious heat avoidance. In this review, we discuss the evidence for a role of TRPA1 in the control of rhythmic behaviours in *Drosophila melanogaster*. Activity levels during the afternoon and rhythmic temperature preference are both regulated by TRPA1. In contrast, TRPA1 is dispensable for temperature synchronisation of circadian clocks. We discuss the neuronal basis of TRPA1-mediated temperature effects on rhythmic behaviours, and conclude that they are mediated by partly overlapping but distinct neuronal circuits. We have previously shown that TRPA1 is required to maintain siesta sleep under warm temperature cycles. Here, we present new data investigating the neuronal circuit responsible for this regulation. First, we discuss the difficulties that remain in identifying the responsible neurons. Second, we discuss the role of clock neurons (s-LNv/DN1 network) in temperature-driven regulation of siesta sleep, and highlight the role of TRPA1 therein. Finally, we discuss the sexual dimorphic nature of siesta sleep and propose that the s-LNv/DN1 clock network could play a role in the integration of environmental information, mating status and other internal drives, to appropriately drive adaptive sleep/wake behaviour.

## 1. Introduction

**Figure 1:**
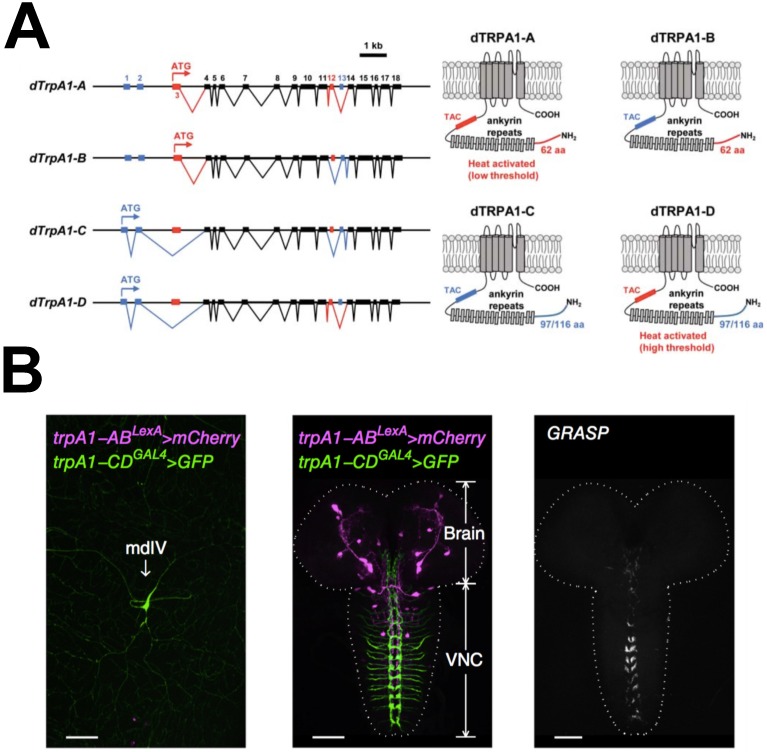
TRPA1 isoforms and their expression pattern in larvae. Taken from [11,15]. (**A**) gene and protein structures of the four TRPA1 isoforms [15]. *trpA1* gene structures with exon numbering are shown on the left. The four isoforms originate from the use of an alternative translational start site (isoforms A/B use ATG in exon 3, red; isoforms C/D use ATG in exon 1, blue) and alternative splicing of exon 12 (included in isoforms A/D, red) and 13 (included in isoforms B/C, blue). TRPA1 protein structures are shown on the right. N-terminal region encoded by exon 3 is drawn in red (A/B), N-terminal region encoded by exon 1–2 is drawn in blue (C/D). TRP ankyrin cap (TAC) region between ankyrin repeats and first transmembrane domain encoded by exon 12 is drawn in red (A/D), the TAC region encoded by exon 13 is drawn in blue (B/C). Presumably, TAC is the element that confers temperature sensitivity to isoforms A/D, whilst the N-terminal region affects their activation threshold and responsiveness to the rate of temperature change [7,11]; (**B**) *trpA1-CD^GAL4^*- and *trpA1-AB^LexA^*-expressing neurons are distinct cell populations that possibly form synapses in the ventral nerve cord (VNC) and may function in a common neuronal circuit [11]. The *trpA1-CD^GAL4^* reporter is expressed in multidendritic neurons (md neurons) in the larvae body wall, while *trpA1-AB^LexA^* is expressed in the larval brain and VNC. Green Fluorescent Protein reconstitution across synaptic partners (GRASP) signal indicates that the two cell populations are in close proximity in the VNC.

Transient receptor potential channels (TRP channels) are variably selective cation channels that consist of six transmembrane spanning domains and are permeable to Ca^2+^ and Na^+^ ions. This family of ion channels is highly conserved between species. TRP channels function in sensory neurons where they detect physical and chemical stimuli. They have been associated with a wide variety of sensory inputs—including vision, touch and thermosensation [1]—and often respond to stimuli of multiple sensory modalities. This is also true for the *trpA1* gene, that encodes a thermosensitive TRP channel (Figure 1A). In *Drosophila*, TRPA1 mediates aversion of chemical stimuli, like the insect repellents citronellal [2] and aristolochic acid [3], and the active component of wasabi, allyl isothiocyanate [4]. In addition, it has been implicated in the avoidance of UV light [5,6] and harsh mechanical and light stimuli [7,8].

In addition to chemical, mechanical and light sensation, TRPA1 is best known for its function in thermosensation. The TRPA1 channel is directly activated by warm temperatures and was in fact the first temperature sensitive channel identified in invertebrates [9]. When *Drosophila* TRPA1 is ectopically expressed in *Xenopus* oocytes or *Drosophila* S2 cells, its activation threshold is between 24 °C and 29 °C [9] or between 30 °C and 36 °C, depending on the TRPA1 isoform [7,10,11] (Figure 1A). In vivo studies have shown that at temperatures below these activation thresholds, indirect activation of TRPA1 happens in a cascade downstream of G_q_ and Phospholipase C (PLC) [3,12]. It has been suggested that both activation mechanisms depend on mechanical deformations of the cell membrane [13,14].

As a small poikilotherm, *Drosophila melanogaster’s* internal body temperature rapidly equilibrates with the environment. Responses to environmental temperature and temperature navigation are therefore extremely important for the proper regulation of physiological processes and for survival. The TRPA1 channel plays a role in these behavioural responses. It functions in the selection of preferred temperatures and the avoidance of noxious heat, which has been extensively described (see [15,16] for reviews). In this review, we shortly discuss the role of TRPA1 in temperature sensitive behaviour, and then review the evidence for a role of TRPA1 in the control of rhythmic behaviours in *Drosophila melanogaster*. These include circadian behaviours, rhythmic temperature preference and sleep. We discuss and compare the neuronal circuits that control these behaviours to get a better insight in TRPA1-mediated effects of temperature on rhythmic processes.

## 2. TRPA1 in Temperature Related Behaviours

**Figure 2:**
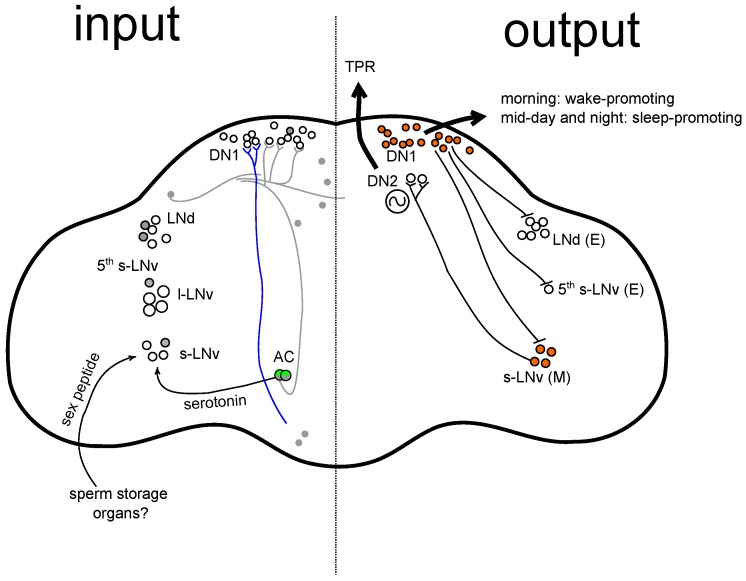
TRPA1 and clock circuits in the *Drosophila* brain that regulate rhythmic behaviours. The left hemisphere shows sensory input pathways to the brain. Right hemisphere shows processing and output that controls rhythmic behaviours. Clock neurons and Anterior Cell (AC) neurons are labelled. Green, TRPA1 expression in AC neurons [19]; grey, *trpA1^SH^-gal4* expression potentially in some clock neurons and in *trpA1^SH^-gal4^+^* cell bodies that are not clock neurons [23]; blue, *ppk-gal4* expression [24]; *trpA1^SH^-gal4^+^* and *ppk-gal4^+^* neurons contact DN1 clock neurons [24]; small arrows, input (via neurotransmitter or neuropeptide) to small lateral ventral neurons (s-LNv) [25,26]; oscillation, the number of s-LNv/DN2 contacts fluctuates throughout the day to regulate temperature preference rhythm (TPR) [26]; bar-headed arrows, DN1 inhibit morning (M) and evening (E) clock cells to regulate siesta sleep [27]; orange, a functional circadian clock in the DN1 or s-LNv is sufficient for siesta during warm temperatures [28].

The *Drosophila* TRPA1 channel has been shown to function in various temperature related behaviours, over a wide range of environmental temperatures. First, TRPA1 plays a role in thermotaxis. Rosenzweig et al. [17] were the first to identify that TRPA1 is required for the avoidance of elevated temperatures (≥35 °C) along a temperature gradient, in all larval stages. The authors showed that a few TRPA1-expressing neurons in the larval brain were involved in this behaviour [17], but the exact neuronal circuit had not been unravelled. In addition to the avoidance of warm temperatures, TRPA1 acts in temperature discrimination within the comfortable range (18–28 ${}^{\circ }$C). Whilst young larvae prefer a temperature around 24 °C, mid- and late-third-instar larvae switch their preference to a temperature of 18 °C [18]. The selection of preferred 18 °C over suboptimal temperatures (19–28 °C) requires TRPA1 [12,18]. In adults, TRPA1 is necessary for the selection of preferred temperatures as well [19]. Adult flies that carried a mutation in *trpA1* or had TRPA1-expressing neurons ablated failed to avoid warm temperatures (28–30 °C). The authors showed that TRPA1 functions in anterior cell neurons (AC neurons), where it possibly acts as a direct warmth sensor (Figure 2). Interestingly, adult temperature preference is a rhythmic process, intuitively coined temperature preference rhythm (TPR) [20]. The neuronal circuits underlying TPR will be discussed in Section 3.5.

In addition to a role in regulating preferred physiological temperatures, TRPA1 plays a role in the avoidance of noxious heat, called thermal nociception, in both adults and larvae [7,21,22]. Using *trpA1* mutants, Neely et al. [21] showed that adult flies failed to avoid hot temperatures of 46 °C when TRPA1 function was lost. Larvae lacking TRPA1 had a reduced response to mechanical touch with a 46 °C probe. The authors showed that TRPA1 acts in md neurons to regulate thermal nociception in larvae. This was a surprising finding, since existing *trpA1* reporters were not known to be expressed in md neurons. Following up from this work, Zhong et al. [7] revealed the existence of two novel TRPA1 isoforms, TRPA1-C and TRPA1-D (Figure 1A). The authors created the transgenic reporter *trpA1-C/D-gal4* using *trpA1-C/D* promoter elements to drive *gal4* expression, which revealed that the TRPA1-C and TRPA1-D isoforms are specifically expressed in md neurons [7]. This was later confirmed by Luo et al. [11] with a different reporter, *trpA1-CD^GAL4^*, a GAL4 knock-in which deletes 723 nucleotides spanning the *trpA1-CD* start codon (Figure 1B). Zhong et al. [7] confirmed that TRPA1-C was the isoform that mediates thermal nociception in larval md neurons. In addition, Zhong et al. [7] showed that only TRPA1-A and TRPA1-D isoforms are directly temperature sensitive due to the presence of a temperature responsive element, which is alternatively spliced between isoforms A/D and B/C (Figure 1A).

A recent study showed that larvae respond to the rate of temperature change in addition to absolute temperatures, and that this behaviour is mediated by the TRPA1-A isoform [11]. The fact that larvae can sense the rate of temperature change allows them to rapidly escape when temperatures quickly rise to noxious levels. The authors showed that brain lateral posterior neurons (BLP neurons) serve as cell autonomous thermosensors in the larval brain, and that activity of the TRPA1-A channel within these cells is regulated by absolute temperature as well as the rate of temperature change. It would be interesting to know if the adult AC neurons respond to the rate of temperature change similar to the larval BLP neurons. The existence and identity of BLP neurons in the adult stage has not been characterised. Finally, Luo et al. [11] propose that TRPA1-C in peripheral cells and TRPA1-A in the brain may act in a common neural circuit to control the rolling behaviour in response to noxious heat in larvae (Figure 1).

## 3. TRPA1 in Rhythmic Behaviours

### 3.1. TRPA1 and Circadian Clock Entrainment

The role of TRPA1 in synchronisation of the circadian clock to temperature cycles is somewhat controversial. TRPA1 had been implicated in synchronisation of locomotor behaviour to temperature cycles (TC) [29]. Lee and Montell [29] found that *trpA1* mutants require an extra day to stably synchronise their activity to shifted TC of 29 °C:18 °C (18 h warm:6 h cold). In addition to this subtle phenotype, the authors found a slight phase advance of the temperature entrained activity in *trpA1* mutant flies. In response to this initial publication, the role of TRPA1 in circadian clock synchronisation to temperature cycles was more thoroughly investigated [23,30]. Two independent studies investigated the locomotor activity patterns of *trpA1* loss-of-function mutants and *trpA1* knock-down under rectangular temperature cycles in constant darkness (DDTC) (DDTC 25 °C:16 °C, DDTC 29 °C:20 °C and DDTC 29 °C:21 °C). Both studies concluded that *trpA1* is not required for behavioural synchronisation to shifted temperature cycles [23,30].

Taken together and strictly speaking, it seems that TRPA1 is not required for synchronisation of the circadian clock to temperature cycles [23,30], although a minor contribution cannot be excluded [29]. The receptors and circuits controlling synchronisation of the circadian clock to temperature cycles are largely unknown. Work from our laboratory has shown that peripheral information from the chordotonal organs (cho) is required [31] and that the *Ionotropic Receptor 25a* and probably also the TRP channel *pyrexia* act within cho to mediate specific aspects of temperature synchronisation [32,33]. Novel temperature receptors and the pathways that feed temperature information to the circadian clock in the brain remain to be identified.

Although TRPA1 is not required for synchronisation of the circadian clock to temperature cycles, it has been shown convincingly that TRPA1 plays a role in the modulation of various circadian behaviours in response to temperature. This will be discussed below.

### 3.2. TRPA1 and "Siesta" under Physiological Warm Temperatures

When exposed to 12 h:12 h light dark cycles (LD) at constant temperature, *Drosophila melanogaster* display bimodal behaviour with characteristic morning and evening activity peaks (M and E peaks) that anticipate light transitions [34,35]. The effect of ambient temperature on these behavioural rhythms was first described by Majercak et al. [36]. At warm physiological temperatures (29 or 30 °C), the M and E peaks advance and delay their phase, respectively, which effectively reduces the amount of activity during midday. This inactivity during midday is aptly referred to as the “siesta”. The temperature dependent changes in behaviour become apparent when observing locomotor activity patterns, but can also be visualised by measuring sleep across the day. It was shown that low locomotor activity levels during the siesta in LD reflect a sleep-like state [37].

When investigating the role of TRPA1 in circadian clock entrainment to temperature cycles (DDTC 29 °C:20 °C), it became apparent that *trpA1* loss-of-function mutants could synchronise their behaviour with temperature cycles, but were much more active during midday at 29 °C [23,30]. The phase of M and E peaks was not altered by loss of TRPA1 [23]. Instead, TRPA1 was required to repress locomotor activity during midday at 29 °C: 20 °C [23,30]. In line with findings by Cao and Edery [37], we have shown that loss of siesta in *trpA1* mutants is caused by a lack of daytime sleep [30]. Taken together, under constant dark conditions and warm temperature cycles, TRPA1 is required to suppress activity during midday, which seems to work via the modulation of activity or sleep levels.

Das et al. [23] extended these findings by showing that TRPA1 modulates activity/rest rhythms also in a light dependent manner. The authors showed that TRPA1 is required for temperature dependent phasing of M and E peaks during LD at warm constant temperatures of 30 °C. In addition, flies subjected to TC in constant light conditions require TRPA1 for proper phasing of the E peak [23]. In both cases, the phasing of activity peaks will affect the amount of midday activity and thereby regulates the siesta. Interestingly, flies are able to suppress midday activity under these conditions, even though they lack TRPA1 expression. This is in contrast with the role of TRPA1 in maintaining siesta during warm TC in constant darkness. Taken together, under conditions where light is present, TRPA1 is required to phase M and E peaks, which seems to work via the modulation of timing rather than regulating levels of activity or sleep.

The phasing of M and E peaks at different ambient temperatures is at least partially dependent on the temperature-sensitive splicing of a 3${}^{\prime }$-terminal intron of the clock gene *period (per)* [36,37,38]. The intron is not efficiently removed at warmer temperatures, which reduces accumulation of *per* mRNA and effectively leads to a delayed E peak and increased siesta. Inefficient splicing at warmer temperatures has been coupled to an increase in sleep during the day, independent of the circadian clock [37]. In contrast, Parisky et al. [28] have recently shown that the effects of temperature on sleep during the day is dependent on the circadian clock, and depends on the expression of PERIOD (PER) in a subset of clock neurons. This raises the possibility that TRPA1 effects on M and E peaks are also clock dependent. In addition, it suggests that TRPA1 effects on the siesta may depend on temperature-sensitive splicing of *per* in clock neurons. The “TRPA1 circuits” and “clock circuits” regulating siesta sleep in response to temperature will be discussed in detail in Section 4 and Section 5.

### 3.3. TRPA1 and "A peak" under Noxious Hot Temperatures

In parallel to studies using rectangular TC, the role of TRPA1 in modulating activity/rest rhythms has been studied in more natural like conditions [39,40]. Locomotor activity profiles of flies measured in the wild or in semi-natural conditions that simulate hot summer days display a novel behaviour that was never observed in laboratory studies before. In addition to the well defined M and E peaks [34,35], these conditions elicit an afternoon activity peak (A peak) [41,42]. Interestingly, Green et al. [39] and Das et al. [40] show that TRPA1 is required to induce the A peak.

The A peak has been shown to be largely clock independent, since it is still present in flies that do not produce the clock proteins PER or TIMELESS (TIM) (*period^01^* and *timeless^01^* mutants) [39,41,42]. In addition, the studies performed by Das et al. [23] have shown that the A peak coincides with T_max_, regardless of the time of day or the time of maximum light intensity. Together, these results suggest that the A peak is a direct response to hot temperatures rather than a clock regulated behaviour. It has therefore been suggested that flies become active in order to escape the high temperatures [40,43], which would be consistent with the role of TRPA1 as a noxious heat sensor in adults [21]. Regardless of whether the A peak is an escape response or not, it has been shown convincingly that the increased activity during the afternoon is not a methodological artefact but is a real behavioural response [39]. If TRPA1 effects on the A peak are largely clock independent, but rather a direct responses to the temperature, this could also explain why these effects are not modulated by light.

It was shown that various *trpA1-gal4* drivers report expression in a subset of clock neurons (see Table 1 and Figure 2). However, TRPA1 antibody does not stain clock neurons [19], which leaves the presence and potential role of TRPA1 in clock neurons uncertain. Green et al. [39] and Das et al. [40] asked the question whether TRPA1 is required in clock neurons to regulate the A peak. Both studies used RNAi induced knock-down of *trpA1* in a subset of neurons to test this hypothesis. First, they showed that knock-down of *trpA1* in all neurons or in *trpA1-gal4^+^* neurons (using *elav-gal4* and different *trpA1-gal4* drivers) was sufficient to eliminate the A peak. In contrast, knock-down of TRPA1 in all clock neurons (using *tim-gal4*) was not sufficient to eliminate the A peak [39], nor was knock-down of TRPA1 in a subset of clock neurons (using *pdf-gal4* and *cry-gal4 39*) [40]. Finally, Das et al. [40] showed that knock-down of *trpA1* in *trpA1^SH^-gal4^+^* neurons that are *cry^-^* (using *trpA1^SH^-gal4/cry-gal80*) again eliminated the A peak. Together, these results indicate that TRPA1 expressed in non-clock cells is sufficient to induce the A peak. The authors showed that expression of the TRPA1-A isoform in *trpA1^SH^-gal4* neurons can rescue the A peak in a *trpA1* mutant background. The TRPA1-D isoform cannot rescue the A peak in these neurons. A role for md neuron-expressed TRPA1-D [7,11] in regulating the A peak has not been investigated.

### 3.4. TRPA1 Has Opposing Effects on Afternoon Activity

Reviewing the role of TRPA1 in temperature regulation of activity/rest rhythms, it becomes apparent that TRPA1 has opposite effects on afternoon activity depending on the experimental paradigm that is used; it reduces activity under physiological warm temperatures (Section 3.2) but induces activity during hot (semi-)natural summer days (Section 3.3). The TRPA1-dependent increase in afternoon activity in response to (semi-)natural summer conditions (A peak) has been observed in constant dark, LD and constant light conditions [40], meaning that light cannot cause the opposite effects of TRPA1 on afternoon activity. Das and Sheeba [44] have recently suggested that these opposite effects are caused by the use of rectangular versus ramped temperature cycles. However, this is not the only difference between the protocols. The natural and semi-natural conditions simulate hot summer days that reach a maximum temperature of 32–35 °C, which is much higher than the rectangular paradigms go (29 °C). In fact, experiments performed by Green et al. [39] have shown that the A peak occurs using gradual temperature cycles of 25–35 °C but not using gradual temperature cycles of 20–30 °C. In addition, Das et al. [40] have shown themselves that the A peak occurs using gradual temperature cycles with a T_max_ of 32 °C, but not with a T_max_ of 28 °C. These data favour the hypothesis that the switch point of TRPA1 dependent modulation of afternoon activity may be depending on environmental temperature, with the occurrence of siesta below 30 °C and the occurrence of an A peak above 30 °C. The ultimate proof of this hypothesis would be the occurrence of the A peak during rectangular cycles with a warm phase of >30 °C; however, this experiment is problematic since flies will most likely not survive a sustained period of 12 h at such high temperatures. This could also explain why the A peak was never observed in laboratory conditions until semi-natural conditions were used.

These different behaviours, and a seemingly opposite role of TRPA1 at physiological temperatures (<30 °C) versus noxious temperatures (>30 °C) match behavioural observations made by Menegazzi et al. [42]. The authors observed the kind of activity that flies displayed whilst temperature increased from 25 to 35 °C. Under temperatures up to 29 °C, the behaviour of the flies did not change with increasing temperature; they either moved around or were immobile. Around 30–31 °C, all flies became immobile, and at 31 °C and above they became hyperactive [42]. Based on the fact that clock mutants were more temperature responsive than wild-type flies, Green et al. ([39] later showed that isogenised *tim^01^* mutants indeed showed enhanced A peak, but isogenised *per^01^* flies did not), Menegazzi et al. [42] hypothesise that the circadian clock suppresses temperature induced activities, as long as they are within the physiological range. When temperatures become noxious, this clock-mediated inhibition is released, and flies become hyperactive. Work from De et al. [45] supports the idea that temperature is more instructive for behaviour during unfavourable (hot) conditions. The authors showed that the timing of adult emergence in *Drosophila melanogaster* was correlated with light under moderate environmental conditions (July month in Bangalore, wet and cool with temperatures between 20–26 °C) but switched to a correlation with environmental temperature and humidity under harsh environmental conditions (April month in Bangalore, hot and dry with temperatures between 20–35 °C). It seems possible that TRPA1 is the temperature sensor that is required for temperature induced behaviours, which are under clock control below 30 °C so that activity is suppressed, but are not clock-controlled at 30 °C or above, so that activity is induced.

### 3.5. TRPA1 and Temperature Preference Rhythm

Temperature preference and TPR are mediated by TRPA1 expression in AC neurons [19,26] (Figure 2). Preferred temperature is relatively low at night and in the morning (24–26 °C), increases throughout the day (25–27 °C), and reaches its peak (26–27 °C) in the evening just before lights off [20]. In a successful attempt to dissect the neuronal circuits controlling TPR, the Hamada laboratory found that the AC-sLNv-DN2 neuronal circuit plays an important role to properly set temperature preference in the hours before dawn (ZT22-24) [26] (Figure 2). The serotonergic AC neurons talk to the serotonin receptor expressing s-LNv neurons. In turn, the s-LNv neurons activate the DN2 neurons, which are the main circadian clock cells that regulate TPR [20]. Interestingly, the number of direct synapses between s-LNv and DN2 varies throughout the day and reaches its peak in the hours before dawn (ZT22-24), correlating with the temperature preference phenotype observed specifically at this time of day.

Since TRPA1 had been implicated in mediating temperature synchronisation of the circadian clock [29], we investigated if this could work via the same serotonergic AC-s-LNv connection. We found that neither silencing, nor depleting AC neurons from TRPA1, nor downregulating the serotonin receptor in s-LNv had any effect on temperature synchronisation ability of the flies, across the range of preferred temperatures (20–29 °C) [26]. From these data, it followed that temperature synchronisation of the circadian clock and TPR must be controlled by distinct neuronal circuits. These findings are consistent with the conclusions that TRPA1, acting in the AC neurons, is dispensable for temperature synchronisation of the circadian clock [23,30].

## 4. TRPA1 Circuit and the Regulation of Siesta Sleep

In the year 2000, *Drosophila melanogaster* was established as a model to study sleep [46,47]. Since then, there is accumulating evidence that *Drosophila* sleep shares essential features with mammalian sleep [48,49], which makes the use of this model and its genetic tools invaluable to gain a better understanding of the mechanistic and neuronal bases of sleep. It has been shown that the regulation of sleep is under the control of two distinct processes. First, there is the homeostatic sleep pressure that builds up during the day and resets during the night. Second, the circadian clock functions as a gate to make sure that sleep occurs at the right time of day. The interaction between homeostatic and circadian processes has been conceptually visualised as the two-process model of sleep regulation [50,51]. Although the two-process model is still influential and conceptually useful [52], this view of sleep regulation turned out to be too simplistic [49]. The efforts to understand sleep from a mechanistic point of view revealed that sleep is regulated by the circadian clock and homeostat, as well as environmental factors, all via distinct mechanisms [49]. In this section, we review the literature describing the mechanistic and neuronal basis of environmental influence on sleep. Because we identified that TRPA1 is required to maintain siesta sleep under warm temperature cycles ([30], see Section 3.1), we specifically focus on the effects of temperature on sleep behaviour and the potential role of TRPA1 therein. We describe the TRPA1 circuit regulating sleep during warm TC (in DD) and discuss difficulties that remain in identifying the responsible neurons.

Recent studies have shown that temperature affects sleep architecture, mainly the distribution of sleep across the day [24,27,28,37,53]. These studies convincingly illustrate that when temperature increases, daytime sleep increases whilst nighttime sleep decreases, and the total sleep levels per day stay fairly constant. Van Alphen et al. [54] report that *Drosophila* sleep exhibits distinct sleep stages that correlate with differences in brain activity, measured as local field potential (LPF). LPF activity is reduced during sleep overall. Interestingly, LPF is higher during daytime sleep than nighttime sleep, revealing differences in the nature of daytime and nighttime sleep. In addition, behavioural responsiveness of inactive flies to a vibrational stimulus is much higher during the day than during the night, meaning that flies sleep lighter during daytime [54,55], and that “siesta sleep” is different from nighttime sleep. The fact that various factors are affecting day and nighttime sleep differentially [53] suggests that they may be mediated by distinct mechanisms and may serve different functions. The redistribution of sleep that has been reported in response to temperature is therefore not trivial. If daytime and nighttime sleep serve different functions, it seems likely that changes in sleep architecture are the result of complex trade-offs. Both internal and external factors should be sensed, weighted and integrated to result in an optimal behavioural decision. Examples of internal factors are the drive to look for food, mates and egg-laying sites as opposed to the internal sleep drive. This must be placed in the external ecological context, where (amongst other things) seasonal changes and varying predation risks during the course of the day affect survival. We study the effect of daily environmental temperature changes, and try to decipher the role of this external factor in the decision making process about sleep/wake behaviour.

Since we and others found that TRPA1 is required to maintain siesta sleep under warm temperature cycles [23,30], we set out to map the neurons that regulate this behaviour. We asked in which neurons TRPA1 is required to induce siesta behaviour under 29 °C:20 °C TCs and tested the involvement of different candidate neurons:

1. AC neurons: AC neurons are a small set of warm-activated thermosensors. They are often called internal thermosensors since they are the only cells in the adult brain known to be autonomously thermosensitive (Recently, cell autonomous thermosensors that express TRPA1 were found in the larval brain [11], the BLP neurons. The existence and function of these neurons in the adult brain has not yet been characterised), without input from peripheral temperature sensors. AC neurons express TRPA1, which is required for their function in temperature preference behaviour [19,26]. In addition, the AC neurons integrate temperature information from peripheral sensors located in the antennae [56].
2. A subset of non-clock dorsal neurons: Under natural and semi-natural conditions that simulate hot summer days, flies show an A peak that is TRPA1 dependent [39,40]. Interestingly, it has been reported that the A peak is dependent on TRPA1 expression in a small subset of neurons [39] that are located in the dorsal brain [57] but may also include the AC neurons [23]. We will call these neurons “non-clock dorsal neurons”, not to confuse them with the DN clock neuronal groups. After we conducted our studies, personal communication with Charalambos Kyriacou revealed that, contrary to what was reported in [39], knock-down of *trpA1* in this small subset of dorsal neurons (using *trpA1^48951^-gal4*) did not interfere with the A peak. This was due to a labelling error that had occurred between the line *trpA1^48951^-gal4* and another *trpA1^GAL4^* (knock-in) line originally created and used by Kim et al. [3]. In fact, the effect on A peak reported in this study [39] was due to knock-down of *trpA1* in a combination of AC and other non-clock neurons, mediated by the latter *trpA1^GAL4^* (knock-in) driver. This is consistent with later findings by Das et al. [40] that the A peak is regulated by TRPA1 in CRY^-^ *trpA1^SH^-gal4*-expressing cells.
3. Clock neurons: Siesta behaviour under LD cycles is at least partly mediated by temperature sensitive splicing of *per* [36], which directly affects arousal state [37]. In addition, it has been shown that temperature effects on the siesta are clock regulated [28]. The authors showed that PER expression in a subset of clock neurons was sufficient to drive siesta behaviour in response to temperature changes. Together, this raises the possibility that clock neurons (by definition, the neurons that express PER) regulate siesta sleep.
4. A combination of the above.

To target these candidate neurons, we used different *gal4* drivers that are expressed specifically in AC neurons, a subset of the non-clock dorsal neurons, clock neurons, or in a combination of these groups. Table 1 provides an overview of the lines, their expression patterns and relevant references. We used the selected *gal4* lines to drive expression of *UAS-trpA1 RNAi*, which employs RNA interference to knock-down *trpA1* in the candidate cells. We purposely avoided rescue experiments (i.e., starting with a *trpA1* mutant background and ectopically expressing *trpA1* only in a subset of neurons), since ectopic expression of *trpA1*, together with the use of temperature cycles that exceed the thermal threshold of the TRPA1 channel, will result in constitutive activation of the candidate neurons [58], making it difficult to distinguish between endogenous and ectopically expressed TRPA1 functions.

**Figure 3:**
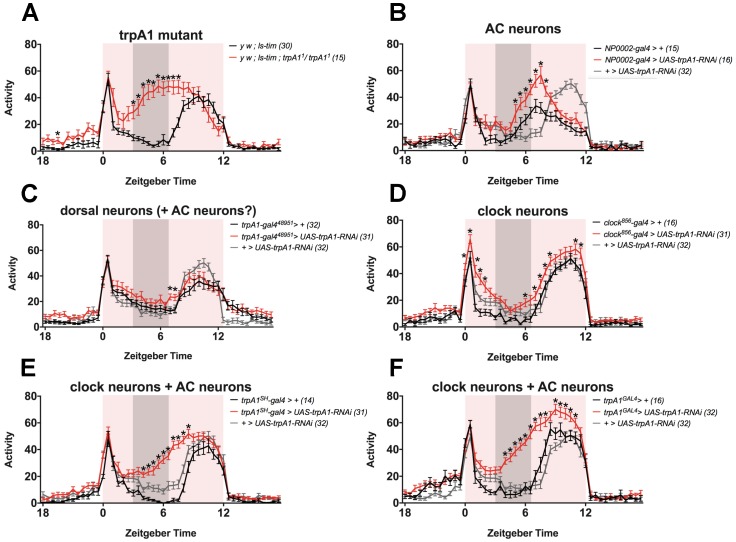
Investigation of the TRPA1 circuit that regulates siesta activity levels during 29 °C:20 °C temperature cycles in constant darkness (DDTC), using *trpA1* knock-down. All figure panels show average activity profiles of indicated genotypes, during the last four days of 29 °C:20 °C TC. Red/white background shadings indicate warm/cold phases. Grey bar (ZT 3-6.5) indicates the siesta. *n* numbers for each genotype are given in the legend. Error bars represent SEM (**A**) activity profiles of wild-type and *trpA1^1^* mutants. Multiple *t*-tests were used to determine significant differences between genotypes at each time point. ∗ means *p* < 0.05; (**B**–**F**) activity profiles of flies with knock-down of *trpA1* in candidate tissues (red) and gal4 (black) and Upstream Activation Sequence (UAS) (grey) control flies. See Table 1 for expression patterns of the *gal4* drivers used. *trpA1^48951^-gal4* is expressed in non-clock dorsal neurons and occasionally in AC neurons. Two-way analysis of variance and Tukey’s multiple comparisons test were used to determine significant differences between genotypes at each time point. ∗ means significantly different (*p* < 0.05) from both *gal4*- and UAS-controls.

The results of these experiments are shown in Figure 3. Figure 3A shows activity profiles of wild-type and *trpA1* mutant flies, confirming the increased activity during siesta when TRPA1 is lacking [30]. Subsequently, Figure 3B–F show the results of *trpA1* knock-down in the different candidate neurons. We can reproduce the phenotype of *trpA1* loss-of-function mutants by knock-down of *trpA1* in a combination of AC neurons and clock neurons, as well as some other cells in the brain (using *trpA1^SH^-gal4* or *trpA1^GAL4^*, Figure 3E,F). When discussing the data further, we focus on *trpA1^SH^-gal4*, since this line has a more limited expression than *trpA1^GAL4^* [23,61,63]. These data show that *trpA1* is required in *trpA1^SH^-gal4*-expressing cells to induce siesta, and that knock-down of *trpA1* in these cells is sufficient to significantly reduce siesta. In contrast, we cannot reproduce the siesta phenotype by knock-down of *trpA1* specifically in AC neurons (using *NP0002-gal4*, Figure 3B). Although the statistics show that experimental flies have increased activity during the late siesta compared to *gal4* and *UAS* control flies, this is the result of an earlier E peak in both *gal4* and experimental flies compared to *UAS* control flies. Finally, we could not reproduce the siesta phenotype by knock-down of *trpA1* specifically in non-clock dorsal neurons and maybe AC neurons (using *trpA1^48951^-gal4*, Figure 3C), or clock neurons (using *clock^856^-gal4*, Figure 3D). When *trpA1* was knocked-down in all clock neurons, activity levels were increased during M peak and E peak, but not during the siesta.

Taken together, these results can be interpreted in two ways:

1. **TRPA1 in AC neurons (*****NP0002-gal4*****), ’dorsal neurons’ (*****trpA1^48951^-gal4*****) or clock neurons (*****clock^856^-gal4*****) may contribute to regulation of siesta, and knock-down solely in these tissues is not sufficient to reproduce the phenotype, presumably because TRPA1 is still functional in other cells.** It is a possibility that TRPA1 regulates siesta in a combination of these neuronal groups, and that knock-down of *trpA1* only reproduces the siesta phenotype successfully when all (or the majority of) these cells are targeted by RNAi. TRPA1 is known to exert its function in AC neurons in the regulation of temperature preference behaviour [19]. Although we observe that TRPA1 is not required in AC neurons for normal siesta under TC (Figure 3B, using *NP0002-gal4*), their known role as temperature sensors and the prominent expression of *trpA1^SH^-gal4* within them, makes it enticing to speculate that AC neurons are indeed part of the circuit that regulates siesta sleep. However, since expression of the *NP0002-gal4* driver has been reported to be very weak [26], we cannot be sure that TRPA1 was sufficiently knocked-down when using this driver. These results should therefore be treated with caution and it remains possible that exclusive TRPA1 expression in AC neurons contributes to the regulation of siesta sleep.
2. **TRPA1 is not required in AC neurons (*****NP0002-gal4*****), ’dorsal neurons’ (*****trpA1^48951^-gal4*****) or clock neurons (*****clock^856^-gal4*****) to induce siesta, and the *****trpA1^SH^-gal4*****-expressing cells that are responsible for siesta regulation are not overlapping with any of these cells.** Based on published expression patterns, this is indeed a possibility [19,23,57,61]. For example, *trpA1^SH^-gal4* expression is reported in four to six cells above the superior arch, and two cells just below it [23], which are not AC or clock neurons [23] and are also not stained by *trpA1^48951^-gal4* ([57] and Figure S3A in [23]) (Figure 2). In addition, *trpA1^SH^-gal4* is expressed in peripheral neurons in the ventral nerve cord (VNC) [61]. *trpA1^48951^-gal4* is expressed in the same region [57], but it has not been characterised if these cells overlap with *trpA1^SH^-gal4* expression. To the best of our knowledge, no peripheral expression studies have been performed with drivers *NP0002-gal4* and *clock^856^-gal4*. It therefore remains an open question if the peripheral expression of *trpA1^SH^-gal4* is specific to this driver or not. Since *trpA1^GAL4^* is expressed widely throughout the brain, it is also conceivable that *trpA1^GAL4^*-expressing cells exist that are not overlapping with AC-, ’dorsal’- or clock-drivers. Taken together, it remains a possibility that *trpA1^SH^-gal4*- and *trpA1^GAL4^*-expressing neurons, which are not AC, ’dorsal’ or clock neurons, are the neurons that regulate siesta behaviour.

These data are consistent with a study by Das et al. [23], describing very similar experiments using a *gal4/gal80* intersectional strategy combined with *trpA1* knock-down. Das et al. [23] first tried to knock-down *trpA1* in all or a subset of clock cells (using *tim-gal4* and *cry-gal4*), which did not reproduce the phenotype. This is consistent with our results using *clock^856^-gal4* (Figure 3D). Regardless of the fact of whether TRPA1 in clock neurons contributes to siesta regulation or not, these results indicate that TRPA1 expressed outside of clock cells is sufficient to induce a normal siesta. To try and exclude the requirement of clock neurons, they next limited the expression of *trpA1^SH^-gal4* to non-clock cells by combining *trpA1^SH^-gal4* with *cry-gal80*. Surprisingly, this also did not reproduce the phenotype. Since TRPA1 is knocked-down in AC neurons and some other neurons, but is intact in clock neurons, this result suggests that knock-down of *trpA1* in non-clock *trpA1^SH^-gal4*-expressing cells is not sufficient to reproduce the phenotype. In other words, the absence of *trpA1* knock-down-and thus the possible presence of TRPA1 - in clock *trpA1^SH^-gal4* cells is sufficient to induce a normal siesta. Since the potential presence of TRPA1 in either clock or non-clock *trpA1^SH^-gal4*-expressing cells is in both cases sufficient to induce a normal siesta, these results support the interpretation that TRPA1 regulates siesta in a combination of clock and non-clock *trpA1^SH^-gal4*-expressing cells, and that the loss of siesta only occurs when TRPA1 is removed from both cell groups.

Antibody staining against TRPA1 has confirmed TRPA1 expression in anterior cell (AC), ventral cell (VC) and lateral cell (LC) neurons in the brain, but never in clock neurons [19]. It seems therefore unlikely that TRPA1 acts in clock neurons, but, how can we then explain the involvement of *trpA1^SH^-gal4*-expressing clock cells? In theory, there are two possibilities:

1. **TRPA1 is actually expressed in clock-cells, but the antibody does not detect it:** Although the TRPA1 antibody is very specific, in theory, it is possible that the antibody is not sensitive enough to detect small amounts of TRPA1 protein. If this is true, it remains possible that TRPA1 is expressed in clock neurons, consistent with the observed expression of *trpA1^SH^-gal4* and *trpA1^GAL4^* in clock neurons.
2. **Unidentical expression patterns of** ***gal4*** **and** ***gal80*** **lines result in false conclusions about the role of TRPA1 in clock cells:** Das et al. [23] used a *gal4/gal80* intersectional strategy to investigate if TRPA1 is functioning in clock and/or non-clock *trpA1^SH^-gal4*-expressing cells. This strategy assumes that the expression patterns of the *gal4* and *gal80* lines are identical. However, it is unknown if the *cry-gal4* and *cry-gal80* expression patterns completely overlap, especially in the periphery. If they don’t target exactly the same cells, this leaves the possibility that *trpA1^SH^-gal4^+^,cry-gal80^+^,cry-gal4^-^* cells exist, in which TRPA1 will not be knocked-down with either *cry-gal4* or *trpA1^SH^-gal4,cry-gal80*. Das et al. [23] suggest this as a possible explanation for the lack of a siesta phenotype in both these genotypes, compared to the observed siesta phenotype with *trpA1^SH^-gal4*. If this is indeed true, this would mean that TRPA1 functions in non-clock and CRY^-^ *trpA1^SH^-gal4*-expressing cells (that are targeted by *cry-gal80* but not *cry-gal4*). To be complete, since *trpA1^SH^-gal4* is expressed in the peripheral nervous system [61], these cells don’t have to be located in the brain but can also be peripheral.

It is difficult to distinguish between these possibilities, and it remains an open question if TRPA1 is expressed in clock neurons and whether it acts within them to regulate siesta under DDTC 20 °C:29 °C. However, regardless of this, it is becoming clear that regulation of siesta sleep in response to temperature is controlled by clock neurons. These “clock circuits” will be discussed in the next Section 5.

## 5. Clock Circuit and the Regulation of Siesta Sleep

There are three recent studies that implicate the involvement of either s-LNv or DN1 clock neurons in the regulation of siesta sleep by temperature [24,27,28] (Figure 2). Lamaze et al. [24] studied the timing of siesta onset in response to temperature. Interestingly, siesta onset was advanced during mild increases in temperature (≤29 °C), which effectively increases the amount of siesta sleep at higher temperatures, consistent with earlier findings [36]. However, the onset of siesta is delayed when temperatures go above 30 °C, which the authors coined prolonged morning wakefulness (PMW). PMW effectively increases the amount of activity in the morning and reduces the amount of siesta sleep. This pattern is reminiscent of the earlier described TRPA1-dependent behavioural switch around 30 °C, with an advancing M peak at warm physiological temperatures and flies becoming hyperactive [42] and displaying increased activity during the afternoon (A peak) [39,40,41] at temperatures above 30 °C. Lamaze et al. [24] found that PMW is also TRPA1 dependent. Thus, the authors identified that TRPA1 regulates the timing of siesta onset, like Das et al. [23] also found under warm LD conditions. Using RNAi knock-down, they showed that TRPA1 is required in two distinct cell populations to regulate PMW; in addition to *trpA1^SH^-gal4*-expressing cells, TRPA1 acts in *ppk-gal4*-expressing cells. It would be interesting to know which TRPA1 isoforms are responsible for the regulation of PMW, and whether distinct TRPA1 isoforms function in peripheral and central circuits, similar to the situation in larvae [11]. Importantly, Lamaze et al. [24] also showed that both *trpA1^SH^-gal4*- and *ppk-gal4*-expressing cells project to the dorsal posterior protocerebrum (DPP) where they contact DN1 clock neurons that are also required for PMW. This study illustrates that two distinct thermosensory circuits contact the DN1s to regulate the timing of siesta onset by the promotion of wakefulness (Figure 2).

Guo et al. [27] performed extensive characterisation of DN1 activity in freely moving animals. The authors showed that DN1 neurons induce siesta during midday via inhibition of other pacemaker cells (the M and E cells) (Figure 2). They propose that DN1s are wake promoting in the morning (like Lamaze et al. [24] also found) and sleep promoting during midday and at night, possibly via the cycling of signalling molecules within the neurons. Importantly, using a calcium assay the authors were able to show that DN1 activity is temperature sensitive. DN1 activity is increased during the day at 30 °C compared to 21 °C, which correlates with the occurrence of siesta behaviour. It is very likely possible that the TRPA1 circuits identified by Lamaze et al. [24] are the neurons that render the DN1s temperature sensitive.

In addition, Parisky et al. [28] studied the contribution of clock neurons in the regulation of sleep by temperature. They found that effects of temperature on daytime sleep but not nighttime sleep depend on circadian clock genes PER and TIM. The authors showed that the presence of a functional clock in either s-LNv or DN1 neurons is sufficient for the increase in daytime sleep at high temperatures (Figure 2). In addition, the authors showed that, in the absence of the circadian clock, the homeostatic sleep drive can also cause an increase in daytime sleep, but this takes several days in contrast to the acute effects of temperature when the circadian clock is intact. Interestingly, the authors were able to show that this regulation of sleep by temperature can be separated from locomotor activity, indicating that sleep is really an independent output and is not downstream of the circadian clock. Furthermore, this study illustrates that regulation of siesta sleep is complex and it highlights the importance of environmental regulation of sleep (as reviewed in Dubowy and Sehgal [49]). Parisky et al. [28] showed that the effects of temperature on sleep are not only regulated by the circadian clock or the homeostat, but they also depend on previous light exposure. They show that, in the absence of light (DD), increased temperature acutely decreases sleep levels, which is independent of the circadian clock and is the opposite of what happens during daytime in LD cycles. The authors propose that a decrease in sleep is the default response to raising temperature (in DD when cycling cues are absent), whilst the presence of light during the day suppresses this default pathway and promotes sleep during daytime. Similarly, the presence of TC can also promote sleep during the day, evidenced by the TRPA1-dependent siesta sleep during DDTC [23,30].

Apart from the TRPA1 thermosensor (maybe acting within clock neurons directly or, more likely, via a TRPA1 dependent circuit), there may be other mechanisms that render clock neurons temperature sensitive. Their temperature sensitivity may be due to temperature sensitive splicing of *per* within them (suggested in Guo et al. [27]), or due to temperature signals received from other (possibly unknown) thermosensors present in the periphery (like *IR25a*, Chen et al. [32]) or brain of the fly (possibly DN2 neurons, suggested in Guo et al. [27]).

## 6. Clock Circuit and Sensory Integration

External temperature cues are integrated with other internal and external cues to drive adaptive sleep/wake behaviour. The study by Parisky et al. [28] nicely illustrates that light and temperature information are integrated, possibly by clock neurons, to regulate daytime sleep. The literature about neuronal control of the siesta shows another interesting example of integration of internal and external cues; we review here a potential role of clock neurons in the integration of environmental information (light and temperature) and mating status.

It is established that female *Drosophila* are more active than males [35]. As expected, *Drosophila* sleep behaviour follows the same sexually dimorphic pattern. It has been shown that the amount of sleep specifically during the day is different between males and females [55,64]. Simply put, males show pronounced mid-day inactivity, the siesta, whilst females do to a lesser extent. In circadian clock research, most of the time only male flies are used. This standardisation makes it easy to compare different studies, and, in our case, it suits our purpose since differences in activity levels, especially those during the day, are more apparent in males than in females. However, from an ecological point of view, it would be interesting to study both males and female flies (e.g., Guo et al. [27]). The fact that males and females display differences in siesta behaviour could inform us about the function of this behaviour in nature.

Interestingly, weakening of the siesta is more pronounced in mated females compared to virgins. The increase of day-time activity after mating has been described as part of the post-mating response in *Drosophila melanogaster* [65] and more recently also in *Drosophila suzukii* [66]. Like other post-mating behaviours, the effect of mating status on daytime activity depends on the transfer of sex peptide (SP) during mating [65]. Interestingly, it has been shown that s-LNv express the sex peptide receptor (SPR) [25] and the authors propose that s-LNv could integrate mating status and sleep pressure (Figure 2).

As discussed above, the regulation of siesta length is under neuronal control. Is this also true for the sex differences observed in this behaviour? As discussed earlier, Guo et al. [27] characterised the role of DN1 activity in regulation of siesta. Strikingly, in addition to temperature sensitivity of DN1s, the authors also showed that DN1 activity is sexually dimorphic. Again, DN1 activity correlates with the siesta phenotypes observed in males and females. Females display lower DN1 activity than males specifically during the day, when DN1 are thought to induce sleep, which correlates with a weaker siesta in females. These data suggest that sex differences in the siesta could indeed be based on sexual dimorphic activity of DN1 clock neurons.

Together these data suggest that the s-LNv/DN1 network could play a role in the integration of environmental information (like temperature and light), mating status and other internal drives like the sleep homeostat, to make a decision whether to promote siesta sleep during the day, or to be active in order to forage and look for egg-laying sites.

## 7. Discussion

In this review, we have discussed the various roles that TRPA1 plays in temperature related behaviours with a main focus on the underlying neuronal circuits. TRPA1 is well known for its roles in temperature preference and noxious heat avoidance, mediated by the central AC neurons and peripheral md neurons, respectively. It was recently suggested that these central and peripheral circuits could act together in a common circuit to provide a dual defence mechanism [11]. Indeed, it has been shown that both central *trpA1^SH^-gal4* and peripheral *ppk-gal4* neurons act together in the temperature-regulation of siesta sleep in adult *Drosophila* (30–31 °C), and both neuronal groups converge on DN1 clock neurons [24]. This is evidence for a TPRA1-DN1 network that regulates siesta sleep during LD cycles, at temperatures around the switch point of 30 °C. In addition, it was shown that siesta sleep is under regulation of the circadian clock, and that an intact clock in the s-LNv or DN1 is sufficient for normal regulation of this behaviour [28]. In the absence of light but in the presence of cycling temperature, it seems most likely that TRPA1 regulates siesta sleep in a combination of clock- and non-clock neurons that are *trpA1^SH^-gal4^+^* (Das et al. [23] and Figure 3 of this review). Although it is enticing to speculate that at least the AC neurons neurons are part of this circuit, the exact identity of the required neurons remains to be identified.

Under noxious temperatures, flies don’t display a siesta during the afternoon but instead display an A peak that is TRPA1 dependent. This behaviour is most likely regulated by non-clock *trpA1^SH^-gal4^+^* neurons [39,40], which fits with the idea that it is a direct temperature response and is independent of the circadian clock. The exact identity of these cells remains to be established, and also whether the non-clock *trpA1^SH^-gal4^+^* neurons that regulate the A peak are the same ones that regulate the siesta under physiological temperatures. It is enticing to speculate that they are, and that the siesta requires TRPA1 in additional cells to allow regulation by the circadian clock.

In addition to the siesta and the A peak, TRPA1 plays a role in regulating TPR. This is mediated by an AC-s-LNv-DN2 network [26]. Both AC neurons and the s-LNv have been suggested to be part of the “siesta circuit”; *trpA1^SH^-gal4* is expressed in AC neurons and potentially s-LNv (Table 1), and a functional clock in s-LNv is sufficient for siesta regulation [28]. It would therefore be interesting to investigate whether the serotonergic communication between AC and s-LNv is also used in the regulation of siesta by temperature. Although similarities between the neural circuits regulating TPR and siesta remain to be established, it is clear that temperature entrainment of the circadian clock does not require TRPA1, nor work via an AC-s-LNv connection [26]. The circuits that are responsible for temperature entrainment of the circadian clock remain largely unknown.

We conclude that TRPA1 plays a prominent role in the regulation of rhythmic behaviours in response to temperature. It is required for TPR, siesta sleep and the A peak, but not for temperature entrainment of the circadian clock. From studying the different neuronal circuits that regulate these behaviours, it seems that the TRPA1 dependent behaviours use distinct but potentially overlapping neuronal circuits. How similar or how distinct these circuits really are remains to be established. The emerging picture is a complex one, with TRPA1 having opposite effects on activity/rest rhythms depending on the absolute temperature, with a switch point around 30–32 °C.

## 8. Methods Accompanying Figure 3

Fly strains. Fly strains and crosses were kept on standard yeast-containing fly food, in a room with programmed 12h:12h LD cycles at constant 25 °C and 60% humidity. *UAS-trpA1 RNAi* (line JF02461) flies were used for *trpA1* knock-down [67], as reported in [39] (from Kyriacou laboratory). *gal4* drivers are listed in Table 1. *NP0002-gal4* and *trpA1^SH^-gal4* (from Hamada laboratory), *trpA1^48951^-gal4* (from Kyriacou laboratory), *trpA1^GAL4^* (from Lee laboratory), *clock^856^-gal4* (from Glossop laboratory). The *trpA1* loss-of-function mutant *y w; ls-tim; trpA1^1^* flies were used as positive controls [12,30]. The *UAS* and *gal4* lines crossed to *y w; ls-tim* flies were used as controls.

Activity monitoring. One- to seven-day-old male flies were loaded in glass tubes, which contained food (2% Bacto-agar, 4% sucrose) on one side, and were plugged with cotton wool on the other side. The glass tubes were loaded in Drosophila activity monitors (DAM2 system; Trikinetics, Waltham, MA, USA) to record locomotor activity. Inside the DAM2 monitor, glass tubes are bisected with an infra-red beam, so that fly locomotor activity can be measured as number of beam crosses. Monitors were placed in light- and temperature-controlled incubators (Percival). Flies were subjected to three days of LD cycles at constant 20 °C and were subsequently subjected to 29 °C:20 °C DDTC, in phase with the preceding LD cycles. After seven days of DDTC, the flies were released into free-running conditions in constant darkness at constant 20 °C. LD and TC conditions were programmed as rectangular cycles of 12h:12 h. Light conditions changed immediately, whist temperature inside the incubator reached the new set point roughly within 30 min. Humidity was increased by placing a water bucket inside the incubator during experiments. In constant conditions, relative humidity was between 75% and 90%. During TC, relative humidity cycled in synchrony with the TC.

Data analysis. Activity data were recorded every minute. Raw data were analysed as total activity counts (beam crosses) per 30 min. After visual inspection of individual actograms, the flies that did not survive until the end of the experiment were excluded from analysis. Microsoft Excel was used for the generation of activity profiles (average of last four days in TC). Statistical analysis was performed using GraphPad Prism 7.

## Figures and Tables

**Table 1:** Expression pattern of selected *gal4* lines used to study the *trpA1* circuit regulating siesta sleep.

Driver	Nature of Gal4	Expression Pattern
*NP0002-gal4*	*gal4* enhancer trap line [59]	Anterior Cell (AC) neurons [26]
*trpA1^48951^-gal4*	short (putative) enhancer fragment of the *trpA1* promoter driving *gal4* [57]	non-clock dorsal neurons [23,57]
		occasionally AC neurons [23]
*clock^856^-gal4*	*clock* promoter fragment driving *gal4* [60]	all clock neurons, but not photoreceptors R1-R8 like *tim-gal4* [60]
*trpA1^SH^-gal4*	*trpA1* promoter fragment driving *gal4* [19]	AC neurons [19,23,61]
		limited brain expression [23,61]
		clock neurons: 1 s-LNv [23], 5th s-LNv [23,62], 2-3 LNd [23,62], 0-1 DN1a [23,62].
		in contrast, [26] don’t find expression in clock neurons.
*trpA1^GAL4^*	GAL4 knock-in into the *trpA1* gene, deleting 185 bp spanning start codon [3]	AC neurons [63]
		wider brain expression [63]
		clock neurons: 5th s-LNv, 3 LNd, 2-3 DN1, 1 DN2, 1 DN3, 3 LPN [29,63].

## Abbreviations

A peak	Afternoon activity peak
AC neurons	Anterior cell neurons
BLP neurons	Brain lateral posterior neurons
cho	Chordotonal organs
DDTC	Temperature cycles in constant darkness
DPP	Dorsal posterior protocerebrum
E peak	Evening activity peak
LD	Light dark cycles
LPF	Local field potential
M peak	Morning activity peak
md neurons	Multidendritic neurons
PER	PERIOD
PLC	Phospholipase C
PMW	Prolonged morning wakefulness
SP	Sex peptide
SPR	Sex peptide receptor
TC	Temperature cycles
TIM	TIMELESS
TPR	Temperature preference rhythm
TRP channel	Transient receptor potential channel
VNC	Ventral nerve cord
